# Role of ILC2 in Viral-Induced Lung Pathogenesis

**DOI:** 10.3389/fimmu.2021.675169

**Published:** 2021-04-19

**Authors:** Wendy Fonseca, Nicholas W. Lukacs, Srikanth Elesela, Carrie-Anne Malinczak

**Affiliations:** ^1^ Department of Pathology, University of Michigan, Ann Arbor, MI, United States; ^2^ Mary H. Weiser Food Allergy Center, University of Michigan, Ann Arbor, MI, United States

**Keywords:** ILC2, RSV, RV, influenza, SARS-CoV-2, asthma, COPD, IPF

## Abstract

Innate lymphoid type-2 cells (ILC2) are a population of innate cells of lymphoid origin that are known to drive strong Type 2 immunity. ILC2 play a key role in lung homeostasis, repair/remodeling of lung structures following injury, and initiation of inflammation as well as more complex roles during the immune response, including the transition from innate to adaptive immunity. Remarkably, dysregulation of this single population has been linked with chronic lung pathologies, including asthma, chronic obstructive pulmonary disease (COPD) and idiopathic pulmonary fibrotic diseases (IPF). Furthermore, ILC2 have been shown to increase following early-life respiratory viral infections, such as respiratory syncytial virus (RSV) and rhinovirus (RV), that may lead to long-term alterations of the lung environment. The detrimental roles of increased ILC2 following these infections may include pathogenic chronic inflammation and/or alterations of the structural, repair, and even developmental processes of the lung. Respiratory viral infections in older adults and patients with established chronic pulmonary diseases often lead to exacerbated responses, likely due to previous exposures that leave the lung in a dysregulated functional and structural state. This review will focus on the role of ILC2 during respiratory viral exposures and their effects on the induction and regulation of lung pathogenesis. We aim to provide insight into ILC2-driven mechanisms that may enhance lung-associated diseases throughout life. Understanding these mechanisms will help identify better treatment options to limit not only viral infection severity but also protect against the development and/or exacerbation of other lung pathologies linked to severe respiratory viral infections.

## Introduction

**Figure 1:**
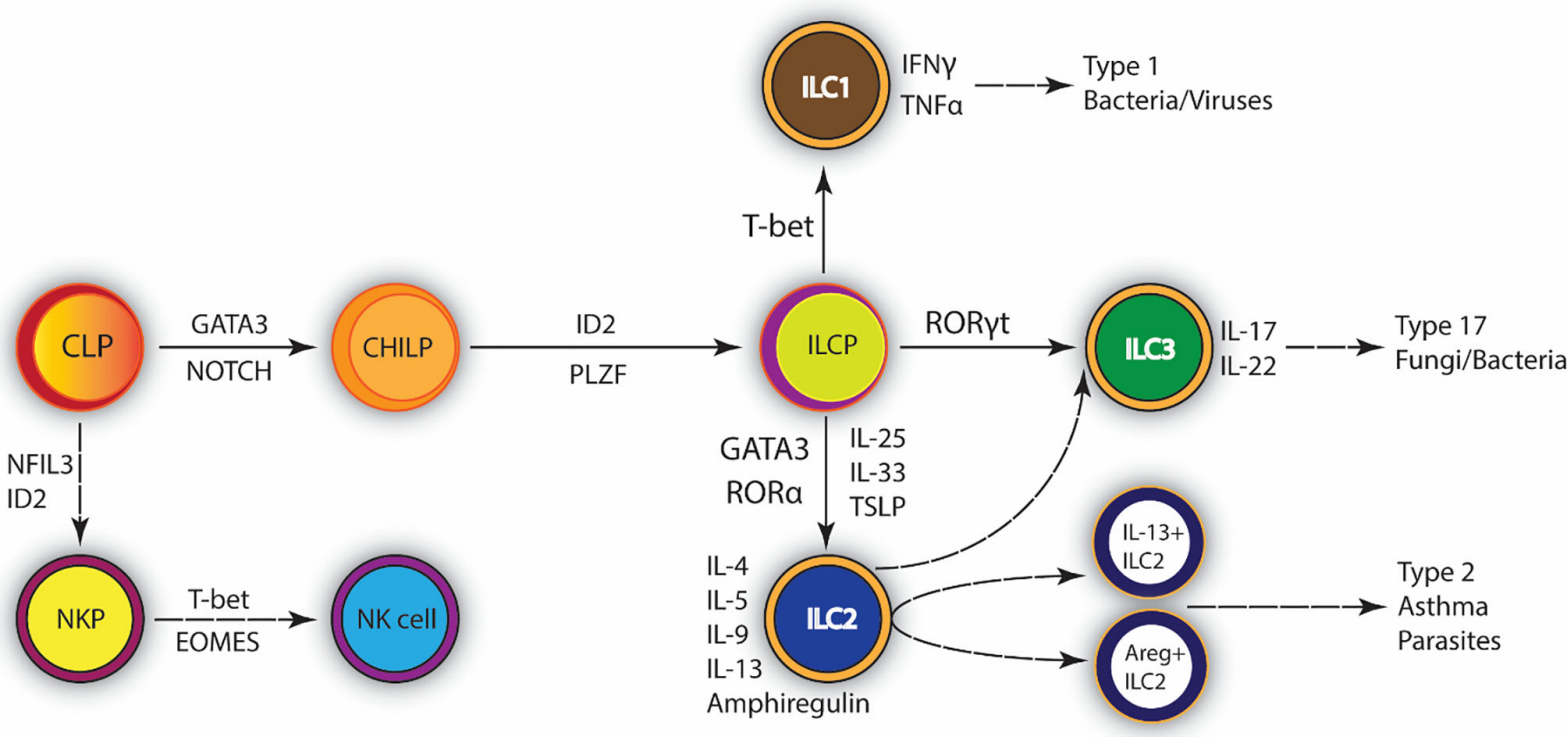
Development of Type 2 Innate lymphoid cells (ILC2). ILC2 promote type 2 inflammation and tissue repair. They are mainly involved in the immune response against extracellular parasites. Common lymphocyte progenitor differentiates into ILC progenitor. GATA-3 is the central transcription factor required for their maintenance and survival. ILC2 are activated by IL-25, IL-33, and TSLP; upon infection they secrete IL-4, IL-5, IL-9, IL-13 and Amphiregulin (Areg). Exaggerated ILC2 immune responses can lead to the development of asthma. CLP, Common lymphoid progenitor; CHILP, Common helper and Innate lymphoid progenitor; ILCP, Innate lymphoid cell progenitor; GATA3, GATA-binding protein 3; ID2, inhibitor of DNA binding 2; NKP, NK precursor; NK, Natural killer cell; PLZF, promyelocytic leukemia zinc finger protein.

Innate lymphoid type-2 cells (ILC2) are a rare population of lymphoid cells that unlike T cells or B cells do not contain an antigen receptor and therefore respond to the immune environment within the tissue compartment. Other ILC populations include ILC1 and ILC3 that differ by their transcriptional regulation and the cytokines that they produce. ILC2 can be differentially characterized as lineage negative, Sca-1+, GATA3+, ST2+, CD25+, ICOS+, and c-kit+ (1, 2). ILC2 differentiation can be driven by innate cytokines, such as TSLP, IL-25, and IL-33, and are characterized by expression of GATA3 and production of cytokines, including IL-4, IL-5, IL-9, IL-13, as well as amphiregulin (AREG). They develop from a common lymphoid progenitor (CLP) in the bone marrow that gives rise to the ILC2 lineage-specific progenitor (ILC2p). The development of this progenitor relies on GATA3 (3–7) and the transcription factor retinoic acid receptor-related orphan nuclear receptor-α (RORα) (7, 8) which is also expressed in common ILC progenitors (7) (**Figure 1**). Mjosberg et al. identified that human ILC2 are also dependent on the transcription factor GATA3 and the innate cytokine, TSLP (5). In addition to inherent transcriptional properties, tissue-specificity has also been shown to play a strong role in ILC2 effector functions (9). Recent mouse studies have indicated that SCF/c-kit activation of ILCp/ILC2 populations within lungs of allergic mice induces important transcription factor expression, ID2 and GATA3, that lead to differentiation and production of IL-5 and IL-13 by ILC2 (10). Thus, the differentiation of ILC2, while not completely defined, appears to require multiple stimuli that likely help to dictate its tissue function under diverse disease responses.

ILC2 are considered the innate counterpart of Th2 cells based on their expression of the transcription factor GATA3 and the production of Th2-type cytokines. It has been suggested that ILC2 are far more potent than CD4+ T cells in their induction of type 2 cytokines; in fact, it is estimated that ILC2 produce 10 times more cytokine than T cells on a per cell basis (11). Innate lymphoid cells play key roles in lymphoid tissue development as well as the initiation of inflammation and more complex roles during the immune response, including the transition from innate to adaptive immunity and chronic inflammation (12, 13). Interestingly, ILC2 may be necessary during early-life for development of the lung and are known to assist in repair and/or remodeling of the lung following injury. However, the numbers increase in the lung during pathogen infections or damaging responses that may require repair of the epithelium. The increased numbers and activation of ILC2 can also lead to chronic disease phenotypes and lead to lung destruction (14–17). TSLP, a cytokine responsible for ILC2 differentiation, appears to be required for initiation/persistence of airway remodeling during chronic allergic asthma (18). The early over-expression of specific cytokines by ILC2 can lead to eosinophilia (IL-5), mucus production (IL-13) and lung remodeling (amphiregulin, AREG). Additionally, studies have suggested that there are subsets of ILC2 that perform different roles in repair and disease with differential contributions to disease phenotypes and ILC2 may have plasticity to allow them to respond appropriately into ILC2 subtypes based upon the immune environment. The effects of early-life pathogenic ILC2 induction may be long-lasting and have an impact on the lung environment well into adulthood as well as contributing to chronic diseases associated with aging. For example, early-life respiratory insults have been linked with an enhanced likelihood of developing asthma and chronic obstructive pulmonary disease (COPD) through long-term lung remodeling (19–21). In addition, ILC2 are known to lead to acute exacerbation of COPD through increased numbers of cells during viral infection leading to enhanced inflammatory damage and in some cases conversion from ILC2 to pro-inflammatory ILC1 (16, 22). Furthermore, as the lung ages, a loss of lung function occurs along with decreased elasticity and changes in the immune response (23) with ILC2 activation leading to enhanced viral-induced damage especially in patients with underlying disease. Interestingly, ILC progenitors in aged mice are increased in bone marrow, but reduced maintenance and function of the cells were observed in the lung (24). Several pulmonary diseases occur during aging and whether the prevalence of ILC2 activity alters the severity of these diseases is presently unclear.

In this review, we will focus on the role of ILC2 during respiratory viral exposures and their effects on the induction and regulation of lung pathogenesis to provide insight into ILC2-driven mechanisms that may enhance lung-associated diseases throughout life. Understanding these mechanisms will be crucial for developing therapeutic as well as prophylactic treatments to protect against initial viral disease as well as the development and/or exacerbation of other lung pathologies linked to severe respiratory viral infections.

## ILC2 in the Developing Lung

Lung ILC2 are tissue-resident lymphocytes that develop early, arising in the fetal mouse lung by embryonic Day 17.5 (25) persisting throughout life (25, 26). Studies have shown that newborn mouse lungs contain very few lymphocytes (27). During the first 3 weeks of life, T, B, and natural killer (NK) cells steadily increase to adult levels, while ILC2 increase more rapidly and reach the adult level by Postnatal (PN) Day 8 and further increase between 10 and 14 days of age when they return to adult equivalent levels (27). The most active phase of lung development (alveolar septation) occurs through the second postnatal week in mice and until 2 to 3 years in humans (28–30) which coincides with the early-life lung predisposition to a type 2 immune environment. ILC2 expansion begins soon after birth when neonatal mouse lungs are activated by IL-33 which persistently alters ILC2 activation and responsiveness throughout life (25, 27, 28, 31–34). Furthermore, Rock and colleagues have identified ILC2 production of IL-13 as being critical for lung regeneration following pneumonectomy lung injury (35). While ILC2 first appear during fetal hematopoiesis, their presence in tissues is established during early postnatal development through rapid expansion, priming, and acquisition of tissue-defining genes (25). This led to the discovery that there are three distinct waves of ILC2 development during early-life that include 1) dispersal into tissues, 2) expansion and activation of tissue-specific transcriptional programs, and 3) homeostatic maintenance with differences in local regulation of survival and turnover throughout life (25). Multiple other studies have shown that many of the ILC2 present in the adult lung were cells that developed during the perinatal period (27, 31, 33). Upon the first breath after birth, IL-33 is spontaneously released from the epithelium which leads to rapid expansion and activation of lung ILC2 (7, 28, 32, 33). In one study, IL-33 production led to a marked increase in ILC2 numbers by PN Day 7 that continued to increase until stabilized by 6 weeks of age (32). Additionally, it was determined that IL-13+ILC2 began to expand at PN Day 3, peaked around PN Day 10, and started to decline at PN Day 14 dependent on IL-33. A similar study by de Kleer et al. supports these findings by showing that type 2 immune cells, including ILC2, accumulate in the lungs at PN Day 3 which leads to enhancement of Th2 responses compared to weanling and adult mice dependent upon IL-33 signaling (28). Importantly, it has been determined that IL-33 activation of ILC2 during the mouse neonatal period has long-lasting effects on ILC2 activation into adulthood which leads to heightened IL-13 production (33). However, it was also observed that a lack of IL-33, using IL-33-deficient mice, had no obvious negative effects on alveolarization (32), indicating that the IL-33 signaling pathway during early-life may be more important for induction of the ILC2-driven type 2 immune response while lung development may be occurring through an alternative or compensatory pathway.

Utilizing an ROR-α lineage tracing model, it has been identified that the neonatal mouse lung consists of distinct ILC2 subsets that have either proinflammatory (Th2 cytokine-expressing) or tissue-repairing (AREG-expressing) properties (7). In addition to these two subsets, it was also shown that the neonatal lung contains an ILC progenitor population (IL-18Rα+/ST2-) that is similar to adult lung ILC progenitors capable of differentiating into multiple different ILC populations. The two distinct neonatal ILC2 effector subsets can be further identified by the genes expressed (7). Both subsets express equal amounts of *Id2, Gata3, Thy1*, and *Il1rl1* (*St2*) but can be further divided into *Il5+/Il13+/Arg1+/Klrg1*+ proinflammatory ILC2 or *Areg+/Icos*+ tissue-repairing ILC2. Interestingly, it has been determined that only the *Il5+/Il13+/Arg1+/Klrg1+* proinflammatory ILC2 subset is dependent on IL-33 regulation (7) and the activation pathway for the *Areg+/Icos+* tissue-repairing ILC2 subset has yet to be elucidated. These findings, along with those by Saluzzo et al. that determined that IL-33 is not required for alveolarization (32), suggest a possible role for *Areg+/Icos+* tissue-repairing ILC2 in the lung developmental process. Furthermore, a recent mouse study indicated that bronchopulmonary dysplasia (BPD), a severe complication of the respiratory system seen in preterm infants, was induced by IL-33/ILC2 signaling caused by an arrest in the process of alveolarization, determining a major destructive role of tissue-resident IL-13+ILC2 in the lung (36). This differentiation could help to identify mechanisms for divergent responses caused by alterations in ILC2 during early-life. It is realistic to assume that in some instances, a loss of AREG+ ILC2 may be detrimental whereby in other cases, the enhancement of IL-13+ ILC2 may be differentially regulated and detrimental. Thus, preferential differentiation of ILC2 that produce IL-13 may be inappropriate and pathologic leading to inflammation and altered lung development. Furthermore, these early developmental changes likely have long-term effects on childhood and adult lung function.

## Role of ILC2 in Th2-Induction During Respiratory Viral Infection

Respiratory infections are responsible for significant healthcare burden throughout the world largely due to the development of lower respiratory tract infections (LRTIs), including bronchiolitis and pneumonia. LRTIs are the leading cause of infectious death in children under the age of five (37). Approximately 80% of LRTI cases are caused by viruses. Among the most prevalent are infections with respiratory syncytial virus (RSV), rhinovirus (RV), and influenza virus. A study evaluating infants hospitalized due to bronchiolitis determined that disease correlated with increased numbers of ILC2 in the nasal passages (38). A mouse study evaluating the immune response to influenza was the first to identify the previously unknown non-T/B cell innate lymphoid “natural helper cell” (ILC2) involvement in respiratory viral infection (39). This latter study determined that IL-13-producing ILC2 were activated through IL-33 production from alveolar macrophages following NLRP3-inflammasome activation by the influenza virus leading to airway hyperreactivity (AHR) (39). Further studies using animal modeling, have discovered that ILC2 are increased in the lungs following other respiratory virus infections, such as RSV and rhinovirus infection (11, 40–48). Also, of note, early in life males are more susceptible to severe disease caused by respiratory viruses compared to females. In the case of early-life RSV, males are hospitalized at 2:1 ratio compared to females due to lower respiratory tract diseases, including bronchiolitis and pneumonia (49). Females tend to have stronger Th1 responses than males, with higher levels of inflammatory markers and viral infection clearance (50, 51) which may induce better protection against infection. Furthermore, clinical studies in children have identified increased atopic diseases in boys compared to girls (50, 52). In support of these findings, blood ILC2 numbers have been shown to be increased in neonates compared to adults with neonatal males having significantly higher levels of ILC2 than neonatal females although no differences were observed in adult men vs woman (53). Interestingly however, testosterone down-regulates ILC2 function, type 2 cytokine production, and expansion during asthma, possibly explaining a reduction in asthma in males post-puberty compared to females (54, 55). Supporting these observations are animal studies that supplement with testosterone to down-regulate ILC2 function and attenuate Th2 cytokine driven allergic disease (54). Thus, age-associated ILC2 function related to early-life viral responses may be mitigated later in life by sex-associated mechanisms in males.

### Respiratory Syncytial Virus

The secretion of innate cytokines, such as IL-25, IL-33, and TSLP, following infection of airway epithelial cells by RSV, leads to the initial activation of the immune response, including the activation of ILC2 (56). While epithelial cells have been directly shown as the main source of these innate cytokines, the brush cells within the lung may also contribute to ILC2 induction in a similar manner as the IL-25 released by tuft cells in the intestine (57) and require further evaluation to expand upon lung ILC2 biology. Importantly, elevated levels of ILC2 were identified in nasal aspirates of infants hospitalized with severe RSV compared to infants with moderate disease and correlated with increased TSLP and IL-33 (58). Stier et al. (11), link the early induction of effector ILC2 to the development of AHR and mucus production associated with RSV through TSLP-driven induction of IL-13-producing ILC2 (11). Administering an anti-TSLP antibody after RSV infection significantly reduced the levels of IL-13-producing ILC2 suggesting a potential therapeutic target. Studies have also shown that RSV driven IL-33-activated ILC2 were crucial for the development of airway inflammation, including eosinophilia and AHR, through ILC2-specific IL-13 production (59). RSV-induced IL-33 appears to be regulated by the type 1 interferon (IFN)/STAT1 pathway with deletion of STAT1 promoting ILC2 activation and Th2 cytokine production (60). Interestingly, age-related IL-33 production was shown to be necessary to induce ILC2 following neonatal but not adult RSV infection that led to Th2-driven immunopathology (41). It has also been identified that ILC2 numbers remain increased in mouse lungs as far out as 4 weeks post-infection following neonatal RSV infection of 7 day old mice along with increased expression of *Il33* and *Tslp*, and the effector cytokines, *Il5* and *Il13* (43, 44). Studies by Fonseca et al. have identified that ILC2 upregulation following RSV in both neonates and adult mice can be driven by uric acid pathways and innate cytokines (e.g. IL-1β, CCL-2, IL-33) that promote ILC2/IL-13-driven Th2 response and immunopathology (43, 45). Finally, ILC2 may regulate RSV-induced CD4+T cell expansion and cytokine expression (especially IL-4/IL-5/IL-13) *via* OX40/OX40L interaction (61). These findings suggest a strong correlation between ILC2 and the development of severe respiratory disease following early-life RSV infection.

### Rhinovirus

In addition to RSV-driven immunopathology, ILC2 have also been implicated in pathologies related to early-life rhinovirus (RV) infection (40, 42, 46–48). Similar to RSV studies, it was determined that rhinovirus infection in neonatal mice led to increased IL-13-producing ILC2 that was not seen in mature mice (47). However in this instance, IL-25 was shown to be the key cytokine responsible (47). Further evaluation of this pathway revealed that following neonatal RV infection both IL-33 and TSLP are required for IL-25-induced ILC2 production of IL-13 leading to immunopathology but TSLP was necessary for maximal ILC2 gene expression even in the presence of IL-25 and IL-33 (40). These results suggest that this group of alarmin cytokines cooperate and regulate each other during respiratory viral infections to expand and fully activate ILC2 populations. One suggested mechanism for the severe disease observed in neonates compared to adults is a lack of a strong Th1/IFN-γ immune response. Immature mice do not induce this pathway whereas adult mice induce strong IFN-γ following RV infection (48). This study showed that administration of IFN-γ immediately following RV infection of neonatal mice attenuated signs of immunopathology suggesting that the lack of IFN-γ is correlated with severe disease. Furthermore, a direct effect of IFN-γ on ILC2 was suggested since there was a reduction in IL-13-producing ILC2 without altering the expression of IL-25, IL-33 or TSLP (48). Importantly, studies that limit ILC2 induction, utilizing an ROR-α inhibitor (SR3335) or genetic deletion of ROR-α, either using complete knockout mice (ROR-α^sg/sg^) or conditional knockout mice to target only ILC2 (*Rora/Il7r^Cre+^*), led to protection from severe disease induced by RV infection (42, 46). Unlike RSV infection (43, 45), RV infection did not drive IL-1β, and neonatal infection in mice lacking IL-1β signaling (NLRP3-/- mice or chemical inhibition of IL-1β) led to enhanced RV-induced Th2 cytokine expression and mucus metaplasia (62). In contrast, another study indicated that NLRP3-inflammasome activity and IL-1β were required for RV-induced airway inflammation and AHR in adult mice (63). The discrepancy between these latter studies may be due to neonatal vs adult mice used in the studies as they also show that adult mice induce IL-1β to a greater extent than neonatal mice following RV infection.

### Influenza Virus

Interestingly, while influenza infection is typically associated with Th1-type immune responses, one of the first studies to identify the role of lung ILC2 during viral-induction of Th2 cytokines was found using influenza (39). The authors identified a lymphoid cell of non-T/B cell origin within the lungs, termed “natural helper cells” which induced IL-13 in response to IL-33 following influenza A infection in mice that led to AHR (39). These results were confirmed by another mouse study that showed ILC2 produced significant Th2 cytokines following pandemic influenza infection (pH1N1) along with increased IL-33 in the lungs and were responsible for the development of AHR, independent of adaptive immunity (64). Another study revealed that c-kit⁺ IL-5-producing ILC2 were activated by IL-33 released from NK T cells and alveolar macrophages and led to eosinophil accumulation (65). A recent mouse study determined that type 1 IFN deficiency leads to ILC2 activation and type 2 immunopathology during influenza A virus infection (66). Furthermore, this study found that type 1 IFN directly negatively regulates both mouse and human ILC2 through regulation of IL-33. Additionally, the authors show that IFN-γ and IL-27 also regulate ILC2 in a STAT1 dependent manner (66) similar to that observed during RSV infection. However, a contradictory study showed that lack of IFN-γ led to protection from lethal infection with pandemic H1N1 and that this was dependent on ILC2 production of IL-5 and AREG which led to increased tissue integrity and reduced immunopathology (67). It is important to note that the latter study did not show differences in IL-13+ ILC2 populations whereas the previous study indicated reduction of IL-13 by the presence of type 1 IFN but not IFN-γ. These studies suggest that type 1 IFN and IFN-γ have different regulatory actions during influenza virus infection and that they appear to modify ILC2 with vastly different results supporting the possibility that there are subsets of ILC2 that are proinflammatory (IL-13+) and another that is responsible for lung repair (AREG+).

### SARS-COV-2 Induced ILC2

Severe acute respiratory syndrome-coronavirus-2 (SARS-CoV-2) is the newly identified β-coronavirus responsible for the pandemic viral pneumonia known as COVID-19. The risk for severe illness with SARS-CoV-2 increases with age, with older adults (>65 years) having five times more probability of developing severe COVID-19 disease (68). It has been reported that there is an increased level of systemic IL-33 in the serum and plasma of severe COVID-19 patients, together with an increased number of circulating ILC2 (69). Furthermore, it has been reported that increased levels of IL-18, IL-13, and IL-6 were increased along with accumulation of ILC2 during COVID-19 that could be linked with the severity of the diseases. A separate study reported increased circulation of ILC2 in moderate but not severe COVID-19 patients suggesting that ILC2 could be differentially regulated based on the severity of the diseases. Low ILC2 could be a marker of severe infection (70). This latter concept may be consistent with reports that ILC2 numbers are decreased by IFN-γ during influenza-induced disease and correlated to more severe disease phenotypes (67). In fact, an original report suggested that ILC2 were responsible for promoting epithelial repair post-influenza infection allowing tissue homeostasis to be reestablished (71). Thus, while ILC2 may provide signals that promote inflammation and pathology, such as IL-13, they likely also have important roles in development and tissue repair early in life and during severe viral-induced epithelial cell damage, perhaps through the differential production of AREG.

## Trained ILC2 Immunity and Exacerbated Lung Disease

### Lasting Impact of Activation of ILC2 in Lung Development and Viral Infections

A strong Th2-immune environment in early-life appears to promote preferential development of Th2 responses that may have long-term consequences on pulmonary diseases (6, 27, 32, 72, 73). Numerous studies suggest that IL-13+ILC2 are responsible for a persistent inflammatory lung environment following early-life viral infection that exacerbates secondary responses later in life (41, 43, 44). Studies have also shown that induction of IL-33 within the lung during early-life leads to enhanced activation of ILC2 which persists well into adulthood (33, 34) and therefore may promote lung structure and functional changes. Furthermore, recent studies have identified that frequent intranasal papain administration, or IL-33 administered in the presence of retinoic acid, may train ILC2 into an “exhausted” phenotype which induces IL-10 that could be harnessed as a potential treatment option for allergic responses to overcome the inflammatory effects of these cells (74).

The phenomenon of “trained immunity” or immunological recall of innate cells, such as myeloid cells (macrophages, dendritic cells, etc.), NK cells and innate lymphoid cells, has been explored in recent years. In the case of ILC2, neonatal “training” has been suggested to impact the activity of these cells during immune responses in adulthood (33, 34, 75). As noted previously, acute expansion and activation of ILC2 occurs shortly after birth and lineage tracing studies determined that 40-70% of these postnatally-derived ILC2 were present in the adult lung (25). This and many other studies have determined that the activity of these cells is dependent on their exposure during the early postnatal period. Intranasal administration of allergen or IL‐33 to mice led to ILC2 expansion and activation in the lung which persisted for up to 1 year after initial administration (34). Importantly, using 5-bromo-2′-deoxyuridine (BrdU)-labeling, the studies show that these were long-lived ILC2 and upon an unrelated allergen challenge or IL-33 administration later in life showed exacerbated responses due to ILC2 production of IL-5/IL-13. Likewise, Steer et al. showed that neonatal mouse lung ILC2 activation by IL-33 has significant effects on ILC2 activity during adulthood (33). Most neonatal lung ILC2 incorporated BrdU and persisted into adulthood. Adult lung BrdU+ ILC2 responded more intensely to IL-33 treatment compared with BrdU– adult lung ILC2. In IL-33-/- mice, lung ILC2 developed normally but because they are not activated during the neonatal period, they have a dampened response in adulthood compared with WT ILC2 (33). Together, these results suggest that activation of lung ILC2 by IL-33 during early-life may “train” ILC2 within the lung that become long-lived resident cells that have stronger type 2 responses to challenges later in life. As indicated in the previous sections, early-life respiratory viral infections likely enhance disease associated “training” that can influence later disease phenotypes (41, 43, 44, 46).

### Viral-Induced Asthma Exacerbations

Asthma often starts during early childhood and according to the Centers for Disease Control, 1 in 12 children (~8%) had asthma in 2017. Asthma exacerbation can be life threatening and often requires hospitalization (76). Viral-induced exacerbation in established pediatric asthmatics is of great concern causing over 80% of exacerbation in these patients with RSV and RV as the most common causes (77). In a cross-sectional, analytical study of children 5-15 years of age that were admitted to the hospital for exacerbated asthma, 20% of those were due to viruses, especially RV and RSV (78). Furthermore, males were hospitalized to a greater extent than females, with boys accounting for ~57% of patients (78) supporting previous findings that males are more susceptible to both viral infection and childhood asthma. The role of ILC2 in asthma and airway disease has been studied recently and reviews describing their role have previously been published (79–82). In severe steroid resistant asthma, ILC2 numbers correspond to the severity of disease exacerbation demonstrating a systemic effect of the disease process (83, 84). The Th2 immune response has been correlated with these exacerbations and since respiratory viral infections enhance Th2 responsiveness of ILC2, it is likely that these cells play a crucial role in exacerbation of asthmatic disease (85). One study first identified that patients with asthma that were subsequently exposed to RV experienced greater RV-induced morbidity and had higher viral loads than their healthy counterparts with increased nasal and bronchial Th2 cytokine levels (86). In addition, asthmatic patients also had increased nasal IL-33 following RV infection. To further explore these findings, human bronchial airway epithelial cells were exposed to RV that led to the induction of IL-33. When the RV-infected epithelial cell supernatants were co-cultured with human ILC2, a strong induction of IL-5 and IL-13 was observed indicating an IL-33/ILC2 pathway activation following RV infection in humans (86).

Mouse models have been developed to begin to unravel the mechanisms of viral-induced asthma exacerbation (87–91). For example, studies have identified that RV leads to the exacerbation of previously established ovalbumin allergy through macrophage/epithelial CCL2-signaling (89) and eotaxin release from macrophages (88). Another study determined that influenza infection potentiates the exacerbation of house dust mite allergic responses (HDM) (91). However, so far, animal studies supporting direct ILC2 involvement in respiratory viral exacerbation of asthma have been limited and require further investigation as this could lead to discovery of new therapeutic targets. In a mouse study evaluating influenza-induced exacerbation of HDM allergic responses, it was determined that CD4+ T cells were the major source of IL‐5 and IL‐13 early during the exacerbation (Day 4) but at later timepoints (Day 7-11), ILC2 contributed more to the total number of IL‐5/IL‐13-producing cells (92). ILC2 appear to be a major source of IL-5 and IL-13 as assessed by the intensity of intracellular cytokine staining (92), indicating higher cytokine production per cell as previously suggested (11). These studies support a role for ILC2 at the time of severe exacerbation as complications and hospitalization during asthma exacerbation often occurs 7-10 days post-viral infection (76). Thus, targeting ILC2 promoting mechanisms may provide a therapeutic option for Th2-associated asthmatic disease. Recent clinical trials using either anti-TSLP or anti-IL-33, which should target ILC2 development and activation, are only now reaching Phase 2/3 human studies but show some promise possibly through regulation of ILC2 function.

### Viral-Exacerbated COPD and ILC2

Chronic obstructive pulmonary disease (COPD) is characterized by severe chronic airway epithelial inflammation that leads to airway remodeling (93), characteristic thickening of the airway wall, increased layers of airway smooth muscle, and increased extracellular matrix (94). It has been reported that worldwide the prevalence of COPD in the population over 40 years is higher in smokers and ex-smokers, and the prevalence increases significantly among persons over 60 years of age (95). While continuous exposure to inhaled irritants that damage the airway function and structure are associated with all causes, the most significant risk factor for COPD is smoking, with ~50% of smokers developing COPD. Respiratory viral infections have been linked with worse outcomes than bacteria exacerbation episodes (96). A recent review of respiratory virus in COPD patients showed that the most common lower respiratory tract virus infections identified in acute exacerbation (AE-COPD) patients were RV (16.39%), RSV (9.9%), and influenza virus (7.83%). Overall, coronaviruses were more frequently detected in the upper respiratory tract than any other virus (97). A recent clinical study indicated increased serum IL-33 levels and numbers of peripheral blood ILC2 in AE-COPD when compared with COPD stable patients and healthy controls (22). When the signature transcription factors were analyzed, they observed increased expression of *Gata3* and *Rora* in the ILC2 sorted from AE-COPD compared with healthy controls and stable COPD patients. Moreover, the expression of these transcription factors was upregulated in the ILC2-sorted cells from stable COPD patients compared with healthy controls (22), suggesting that the proliferation and activation of ILC2 are associated with AE-COPD and that active ILC2 could be involved in the pathogenesis of COPD. While ILC2 have been most notably explored in COPD patients and in animal modeling, ILC3 and production of IL-17 has also been suggested. Importantly, ILC2 exposure to IL-12 during viral exacerbation can mediate conversion to IFN-γ-producing ILC1 correlated with the severity of COPD exacerbations, demonstrating that ILC2 plasticity exists (16).

Lung ILC2-related enzyme arginase 1 (Arg1) is upregulated in asthmatic, idiopathic pulmonary fibrosis (IPF), and COPD patients and is a marker for lung ILC2 (98). Arg1 is part of the L-arginine metabolic pathway and drives collagen synthesis as well as bioenergetic pathways critical for cell proliferation (99). It has been shown that Arg1 ILC-intrinsic deletion abrogated type 2 lung inflammation by decreasing ILC2 proliferation and activation (100). Furthermore, the latter demonstrated that tissue samples from COPD and IPF patients presented ILC2-arginase positive staining, suggesting that ILC2 in COPD and IPF could be targeted with inhibitors of Arg1 to control ILC2-induced disease responses. To understand the role of ILC2 in the development of COPD, an experimental mouse model of COPD (exposure to cigarette smoke for 12 weeks) was performed using ILC2-deficient mice (*Rora^fl/fl^/Il7r^Cre^*). Cigarette smoke-exposed *Rora^fl/fl^/Il7r^Cre^* mice were protected from emphysema development but interestingly presented increased IL-33, IL-13, and ILC2 numbers (101). However, it should be noted that there was no viral infection of these animals and the subsets of ILC2, such as Areg+ILC2, were not examined. Overall, the role of ILC populations in the development or exacerbation of COPD has not been clearly defined and will require future investigation but correlations suggest ILC subsets are involved in COPD pathogenesis, especially AE-COPD groups.

### ILC2 and Idiopathic Pulmonary Fibrosis (IPF)

Interstitial lung diseases can cause significant morbidity primarily in older individuals when accompanied by compromised lung function. Idiopathic pulmonary fibrosis (IPF) is likely the most severe form of interstitial disease. Several risk factors have been linked with the development of IPF, including smoking, environmental inhaled exposures, chronic viral infections, genetics and comorbidities (102). Hallmarks of Aging: abnormal telomere shortening, mitochondrial dysfunction, cellular senescence, impaired autophagy, and epigenetic reprogramming, among others, are suggested to be essential during IPF pathogenesis (103). Furthermore, IPF has been linked with IL-13 and TGF-β production and ILC2 have been recognized as an essential source of these cytokines within the lung (10, 104). Increased levels of epithelial-derived cytokines IL-33, TSLP, and IL-25, critical activators, and recruiters of ILC2, have been detected in lung tissue and/or bronchoalveolar lavage (BAL) fluid of IPF patients (17, 105, 106). Together, these data suggest that ILC2 could play a critical role in the pathophysiology of IPF. A mouse model of bleomycin-induced pulmonary fibrosis showed a similar trend, with increased expression of IL-33 in the lung (107). In this latter study, bleomycin-induced pathology was not affected by deficiency of the IL-33 receptor (ST2) that is associated with ILC2 activity. Interestingly, the delivery of IL-33, using adenovirus-targeted delivery, during bleomycin treatment, showed a synergistic effect on airway inflammation, collagen accumulation, upregulation of heat shock protein 70 (HSP70), TGF-β, IL-6, CCL2/MCP-1, MIP-1α and TNF-α, but did not alter type 2 cytokines (107). Another bleomycin study in mice determined that fibrosis development was dependent on IL-33/ST2 signaling and that the adoptive transfer of ILC2 into the lung during treatment led to enhanced disease (108). In addition, systemic sclerosis patients were found to have increased tissue ILC2 that correlated with both fibrotic skin lesions as well as the presence of interstitial lung disease (109). Acute exacerbation of IPF (AE-IPF) is associated with increased mortality. In a clinical study where AE-IPF patients were tested to identify the pathogen (e.g. virus vs bacteria) involved in exacerbation, viral-positive nasopharyngeal swabs were reported in 60% of these patients (110), suggesting that respiratory virus incidence in the development of AE-IPF is higher than bacterial infection. As described above, RSV and RV immunopathology is highly linked to ILC2 activation and recruitment and therefore, these viruses could promote further acceleration of the fibrotic responses. Other viral infections including herpes viruses and cytomegalovirus have been implicated in the progression of IPF. However, no data have established a link of these latter viral infections with ILC2 biology. Additional investigations into the role of ILC2 in promoting lung remodeling may identify them as having an important role in the progression of interstitial lung diseases. Thus, while a causal effect of ILC2 for the development of pulmonary remodeling has not been established, the ability of ILC2 to produce IL-13 and/or AREG that can promote myofibroblast activation provide a rationale for further investigating their role in these chronic remodeling diseases.

## Strategies to Mitigate ILC2-Induced Pathology

Over the past several years a number of therapeutic targets have been advanced to clinical trials that can impact ILC2 activation, expansion, and mediator release that effect clinical disease. Two targets, TSLP and IL-33, appear to have significant impact on the development of chronic severe asthma responses, especially related to exacerbations. As indicated in the previous pre-clinical research, ILC2 likely play a critical role in not only maintaining a Th2 phenotype in the lung, but also for directing an inappropriate anti-viral response that leads to a worsening of the tissue responses. While the clinical studies have not reported ILC2-associated changes specifically, the biology would predict that there would be a significant effect on ILC2 over time, allowing a more appropriate immune environment. TSLP and IL-33 inhibition may alter ILC2 biology and therefore target their development, however, complementary targeting of ILC2 products may be more successful. The ability to target IL-13 biology has already been shown to be effective in chronic Th2-mediated disease, including asthma, with the IL-4Rα antibody, Dupilumab. While all of these ILC2-related targets hit many aspects of chronic disease biology, their role in long-term disease mitigation likely impacts ILC2 as one of the central components of disease severity. Future targets may depend upon the disease and the pathologic phenotype of the response, such as targeting AREG during chronic remodeling diseases, such as IPF. To properly identify viable ILC2 therapeutic targets, future studies examining the mechanisms of how ILC2 subsets impact the lung environment and how ILC (e.g. ILC1/2/3) differentiate will be defining for individual disease phenotypes.

## Conclusion

**Figure 2:**
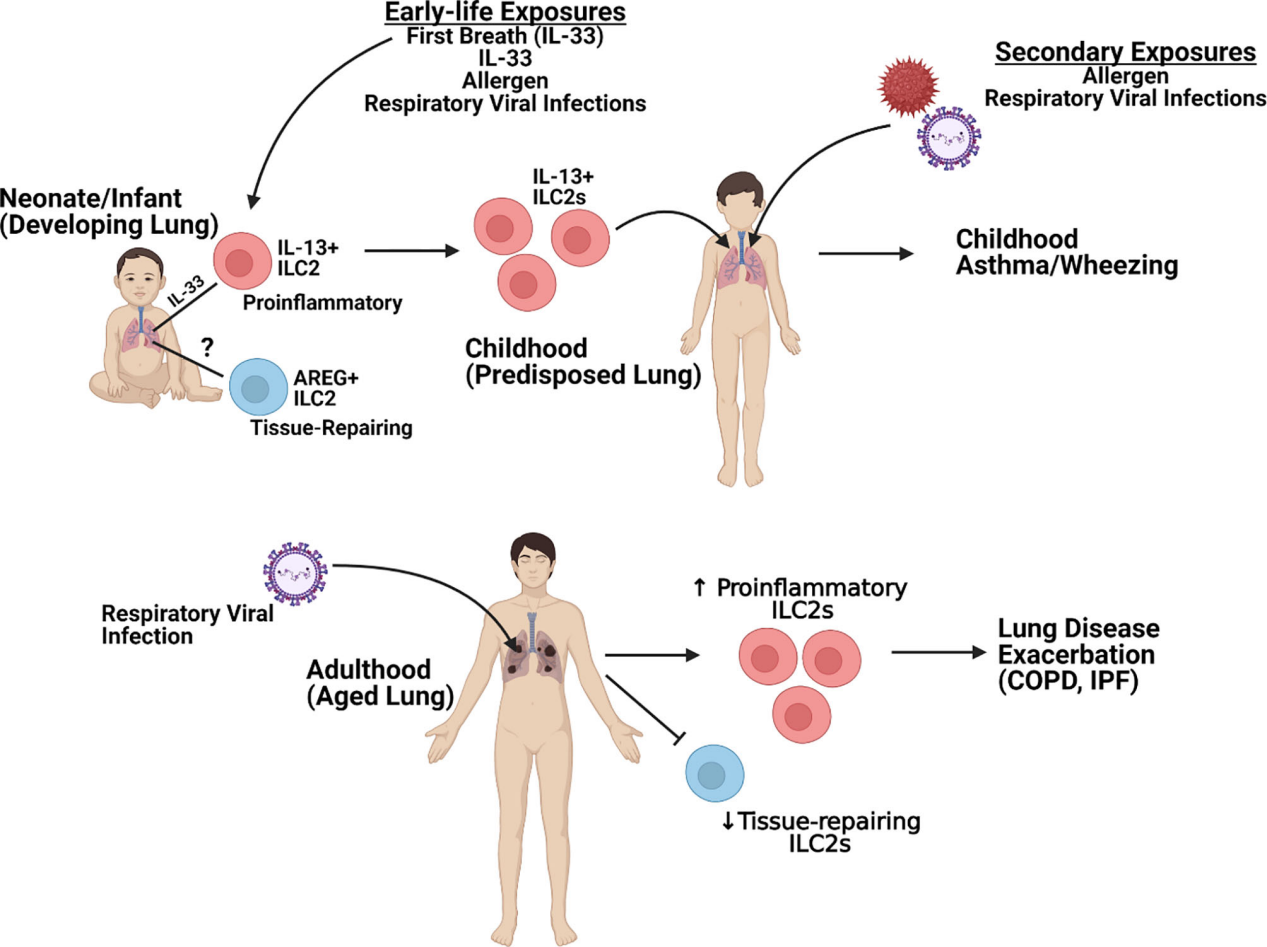
Overview of ILC2-specific Lung Pathogenesis. *Figure created using* Biorender.com.

Severe lung disease induction by respiratory viruses continues to be a significant healthcare burden and cause of morbidity and mortality worldwide. These diseases have been associated with the induction of strong Th2-type immune responses. ILC2 are now recognized as a significant contributor of dysregulated immunopathology within the lung following respiratory viral infection as well as during the pathogenesis of lung diseases, such as asthma. This review has highlighted the role of ILC2 during the development of the early-life lung and how subsets of ILC2 may become persistently altered following early-life infection to alter immune responses to future pathogens as well as how the effect of ILC2 responses on the aged lung later in life leads to enhanced complications (**Figure 2**). On the contrary, ILC2 may also play a protective role during lung disease pathogenesis by maintaining and/or repairing the lung epithelium. Therefore, critical targeting of specific ILC2 subsets [e.g. proinflammatory (IL-13) or tissue-repairing (AREG+)] will be crucial when evaluating potential therapeutic candidates. Elucidation of the mechanisms in which these cells may be damaging and/or protecting the lung immune and structural environment will help identify better treatment options to not only protect against initial disease but also reduce the development and/or exacerbation of other lung pathologies linked to severe respiratory viral infections.

## Author Contributions

All authors contributed to the article and approved the submitted version.

## Funding

This work was supported by National Institutes of Health Grants R35HL150682, R01AI138348, and P01AI089473.

## Conflict of Interest

The authors declare that the research was conducted in the absence of any commercial or financial relationships that could be construed as a potential conflict of interest.
